# Colonization of the Scottish islands via long-distance Neolithic transport of red deer (*Cervus elaphus*)

**DOI:** 10.1098/rspb.2016.0095

**Published:** 2016-04-13

**Authors:** David W. G. Stanton, Jacqueline A. Mulville, Michael W. Bruford

**Affiliations:** 1 School of Biosciences, Cardiff University, The Sir Martin Evans Building, Museum Avenue, Cardiff CF10 3AX, UK; 2 School of History, Archaeology and Religion, Cardiff University, Humanities Building, Column Drive, Cardiff CF10 3EU, UK

**Keywords:** red deer, ancient DNA, colonization, Neolithic

## Abstract

Red deer (*Cervus elaphus*) have played a key role in human societies throughout history, with important cultural significance and as a source of food and materials. This relationship can be traced back to the earliest human cultures and continues to the present day. Humans are thought to be responsible for the movement of a considerable number of deer throughout history, although the majority of these movements are poorly described or understood. Studying such translocations allows us to better understand ancient human–wildlife interactions, and in the case of island colonizations, informs us about ancient human maritime practices. This study uses DNA sequences to characterise red deer genetic diversity across the Scottish islands (Inner and Outer Hebrides and Orkney) and mainland using ancient deer samples, and attempts to infer historical colonization events. We show that deer from the Outer Hebrides and Orkney are unlikely to have originated from mainland Scotland, implying that humans introduced red deer from a greater distance. Our results are also inconsistent with an origin from Ireland or Norway, suggesting long-distance maritime travel by Neolithic people to the outer Scottish Isles from an unknown source. Common haplotypes and low genetic differentiation between the Outer Hebrides and Orkney imply common ancestry and/or gene flow across these islands. Close genetic proximity between the Inner Hebrides and Ireland, however, corroborates previous studies identifying mainland Britain as a source for red deer introductions into Ireland. This study provides important information on the processes that led to the current distribution of the largest surviving indigenous land mammal in the British Isles.

## 1. Introduction

For at least the last 50 000 years, humans have played an important role in faunal redistribution [1,2], with early people moving animals for both food and cultural reasons [3]. In the case of islands that could not have been colonized naturally, wildlife distributions and phylogeographic patterns can inform us about human maritime movements [4]. There is evidence for the maritime colonization of Europe by Neolithic people of the Near East via the Mediterranean [5], with isolated long-distance sea travel around the Mediterranean as early as the Mesolithic [6]. There are fewer such examples of ancient sea travel in northern Europe, although it is thought that humans introduced fur-bearing species to the Scottish Isles from the Neolithic onwards [7] and transported red deer (*Cervus elaphus*) from Britain to Ireland during the Irish Bronze Age [8]. There is also evidence of long-distance movement of wildlife (Orkney vole) by Neolithic people between continental Europe and Orkney [4]. An increased knowledge of wildlife movements during the Holocene can help us to describe the nature of the historical relationship between humans and animals, and to better understand the role that humans have played in shaping evolutionary processes in wildlife throughout history [3].

Red deer are currently one of the most widespread large mammals in Europe, absent only from northern Scandinavia, Finland and Iceland [9]. The species was also distributed widely across western Europe prior to the last glacial maximum (LGM; approx. 27 000–24 000 cal. yr BP), but was confined to southern refugia during glacial periods [10]. European red deer mitochondrial (mt)DNA has been shown to be structured into either three (western Europe, eastern Europe and Mediterranean [11,12]), or four (western Europe, Balkan, Middle East and African [13]) distinct genetic lineages, which are thought to have originated from these southern refugia. In addition to historical climate change, deer are thought to have been subject to human-mediated movement and management throughout Europe for thousands of years [14–16], and records exist of historical introductions into the UK from as far as North America [17]. For example, the population on the Isle of Rùm, Inner Hebrides, is descended from at least four different mainland UK populations, with some individuals clustering with western European red deer while others cluster with deer from Sardinia and North Africa [18]. Additionally, red deer from Norway are genetically closer to Scottish deer than their Swedish and Danish neighbours [19], leading to the suggestion that the Norwegian deer originate from a different source population [20]. Red deer therefore provide an interesting case study for testing the relative contributions of human versus natural post-LGM recolonization events.

Archaeological faunal assemblages within Britain indicate that deer were present on a range of insular locations lying at varying distances from the British mainland. Many of these islands have been isolated since the end of the last glaciation and even today host a restricted range of mammalian fauna. In Scotland, red deer are reported from Mesolithic (7500–5500 cal. yr BP) Inner Hebridean assemblages; however, while there is emerging evidence for Mesolithic settlement on all the outer island groups (e.g. [21,22]), no contemporaneous red deer populations have been identified. Red deer first appear as Neolithic (5500–4500 cal. yr BP) arrivals in the more distant Outer Hebrides and Orkney [23]. As red deer are thought to be able to swim up to 7 km [24] they could move from the mainland to the closer Inner Hebridean islands unaided, and this may explain their early occurrence. Their ability to make the 25 km crossing from Skye to the Western Isles over the Miniches or 16 km across the Pentland Firth to Orkney is unlikely, thus humans probably facilitated their movement. The combination of natural versus anthropogenically mediated colonization makes the Scottish Isles an excellent location for investigating island colonization.

Ancient DNA (aDNA) can give insights into the dynamics of populations that would not be possible to determine from archaeological information or DNA analysis of modern populations alone [25,26]. This is particularly relevant in the case of red deer, due to the complex history of human-mediated translocations [8]. This study uses DNA from opportunistically collected deer bone to provide a unique, serially sampled dataset from a restricted geographical range. This study aimed to describe the genetic diversity of ancient red deer populations on the Scottish islands, and compare this with other contemporary and modern red deer populations to infer details of historical red deer colonization. We hypothesized that the ‘outer isles’ (Outer Hebrides and Orkney) samples would show a subset of the genetic diversity present in the Inner Hebrides/mainland Scotland samples, indicating the colonization of the former from the latter. We use partial mitochondrial DNA (mtDNA) control region sequences isolated from insular and mainland Scottish red deer bone samples from between the Mesolithic and Norse Age to investigate this hypothesis.

## 2. Material and methods

### (a) Samples and study sites

Sampling was focused on red deer derived from a range of Scottish insular and mainland archaeological sites that represent the spatial and chronological distribution of this species over the last 7500 years. We attempted to extract and sequence mtDNA control region fragments of 74 ancient red deer samples from the Scottish mainland (*n* = 7) and islands (Inner Hebrides, *n* = 13; Orkney, *n* = 17; Outer Hebrides, *n* = 37). Material was selected that dated from Mesolithic to Late Norse contexts, located either within a radiocarbon-dated or an artefact-dated secure stratigraphic sequence. Full sample information is given in electronic supplementary material, table S1. Samples were initially collected for a project exploring foodways across the Northeast Atlantic archipelagos [27] and were limited by preservation biases, with the result that few examples of mainland red deer were available for study. The material analysed was selected to minimize the risk of repetitively sampling one individual. Where possible, material derived from sampling from the same side and location of one particular element. Where this was not possible, material was sampled from different archaeological contexts. Antler was not sampled, as shed antler could represent multiple elements from one individual, and was often transported as material for tools and working. Material from mainland sites were obtained from Applecross and Risga (a small rocky island lying 250 m offshore in Loch Sunart). A wider range of insular sites were sampled. Inner Hebridean sites were represented by material from three midden sites on Oronsay, and in the Outer Hebrides, material from both the Lewis Harris and Uist landmasses was sampled. Orcadian material was derived from the smaller islands of Hoy and Westray, as well as Mainland Orkney; both of the former lie less than 5 km across open water from Mainland Orkney and other linking islands.

We included all available and comparable aDNA sequences from previously published studies, including Norway [20], Ireland [8] and Italy [21]. When an age of a sample was estimated in one of these studies, we used the midpoint of this estimate to assign to a time period. Time periods were classified as follows: Mesolithic, 7500–5500 cal. yr BP; Neolithic, 5500–4500 cal. yr BP; Bronze/Iron Age, 4500–1100 cal. yr BP; Norse, 1100–500 cal. yr BP (electronic supplementary material, methods S1). Details of all included haplotypes are given in electronic supplementary material, table S2. Sequences from an additional study that used aDNA sequences [10] could not be included due to limited homologous sequence between the two studies.

### (b) Sample preparation

Bone samples were cut into approximately 0.5 g fragments using a Dremel multi-tool drill. Each fragment was sonicated (Kerry Ultrasonics cleaning bath) in 50% bleach, 100% ethanol and water for 5 min each to remove any external DNA.

### (c) DNA extraction and PCR

Bone fragments were powdered in a freezer mill (Glen Creston Ltd, 6700), using liquid nitrogen. Equipment that came into contact with the sample (plastic tube, and metal hammers and plugs) was thoroughly washed in bleach and UV irradiated for at least 48 h between samples. Powdered bone samples were dissolved via an overnight incubation at 56°C in 15 ml of EDTA (0.5 M), lauroyl sarcosinate (1% w/v) per 1 g of sample weight and 60 µl Proteinase K. DNA was extracted using a Blood & Tissue kit (Qiagen, GMBH, Germany), but starting from the second step (addition of buffer AL). The recommended volume of sample plus EDTA buffer, buffer AL and ethanol was doubled, enabling us to run double the recommended volume of DNA extract through the spin column. We carried out three parallel replicates for each sample. All pre-PCR laboratory work was carried out in laboratories dedicated to aDNA work at Cardiff School of Biosciences.

We used three pairs of PCR primers from Carden *et al*. [8] that amplified short fragments of the mitochondrial control region: RD1F (5′-CCA CYA ACC AYA CRA CAR AA-3′) and RD1R (5′-TTR TTT AYAGTA CATAGT RCATGATG-3′); RD2F (5′-GCC CCA TGCWTATAA GCA TG-3′) and RD2R (5′-CCA TGC CGC GTG AAA CCA-3′); and RD3F (5′- GAT CAC GAG CTT GRT YAC C-3′) and RD3R (5′-TTC AGG GCC ATC TCA CCT AA-3′). These overlapping fragments concatenate to a single 327 bp fragment (position 15 573–15 899, based on complete red deer mtDNA genome, GenBank accession number AB245427). The PCR mix contained 50% (v/v) Multiplex Mix (Qiagen; consisting of Qiagen Multiplex PCR buffer with a final concentration of 3 mM MgCl_2_, dNTP mix and HotStarTaq DNA polymerase), 10% (v/v) Q solution (Qiagen), 1 µg BSA and 2 pmol of each forward and reverse primer. We used 2 µl of DNA extract in reaction volumes of 10 µl. PCR cycling conditions were as follows. RD1: 95°C for 15 m; 10× (94°C for 30 s, 52°C for 90 s, 72°C for 90 s). 50× (94°C for 30 s, 60°C for 90 s, 72°C for 90 s); 72°C for 10 m. RD2 and RD3: 95°C for 15 m; 60× (94°C for 30 s, 60°C for 90 s, 72°C for 90 s); 72°C for 10 m. One PCR reaction was carried out for each of the three DNA extraction repeats, for each sample. Our aDNA protocol is specified in electronic supplementary material, methods S1.

### (d) Molecular analysis

Sequences were aligned to each other and to an outgroup (*Cervus nippon*, GenBank accession number FJ743502) using Sequencher 4.9 (GeneCodes), and then checked by eye. For any given base position, any ambiguous bases (base position with secondary peaks ≥50% of primary peak, in either forward or reverse direction, or any of the overlaps between fragments, or duplicate sequences) were called as an ‘N’. Networks were created using TCS 1.21 [22], and visualized using Cytoscape v. 3.2.1. We created two phylogenies using only the ancient samples, one neighbour-joining tree using MEGA 6 with 1000 bootstrap replicates, and one Bayesian tree using MrBayes v. 3.2.2 [28] with a GTR substitution model and rate variation across sites with a proportion of invariable sites (JModelTest 2.1.7 [29,30]). We also constructed a Bayesian tree of all sequences (modern and old; electronic supplementary material, table S2) used in this study, with the same model of sequence evolution as for the previous tree. Genetic diversity indices (haplotype diversity and F_ST_) were estimated using DNAsp 5 [31] and Arlequin 3.5 [32], respectively.

## 3. Results

### (a) PCR success

PCR success rate was 64.4%, corresponding to 46 samples with sequence data (62.2% ‘sample success’ rate). There were no clear differences when considering age of sample or sampling site: sample success rate ranged from 50.0% to 83.3% across sites, and from 60.0% to 66.7% across sample age (excluding sites and ages with only one sample). Sites and locations for samples that were successfully sequenced in this study are given in table 1. PCR success for each sample is given in electronic supplementary material, table S1.

**Table 1.** Locations and sites of the samples that we successfully sequenced in this study. The sites are shown in figure 1, and more detailed descriptions of each sample are given in electronic supplementary material, table S1.

location	sites	no. samples	haplotypes
mainland Scotland	Applecross Broch, Risga	4	Hap007, Hap010
Inner Hebrides	Caisteal Nan Gillean II, Cnoc Sligeach, Priory Midden	9	Hap010
Outer Hebrides	Northton, Bornais, Baile Sear	22	Hap009, Hap011, Hap014, Hap015, Hap017, Hap018, Hap019, Hap020
Orkney	Ness of Brodgar, Mine Howe, Links of Noltland	11	Hap008, Hap011, Hap012, Hap013

### (b) Ancient red deer genetic diversity

**Figure 1.**
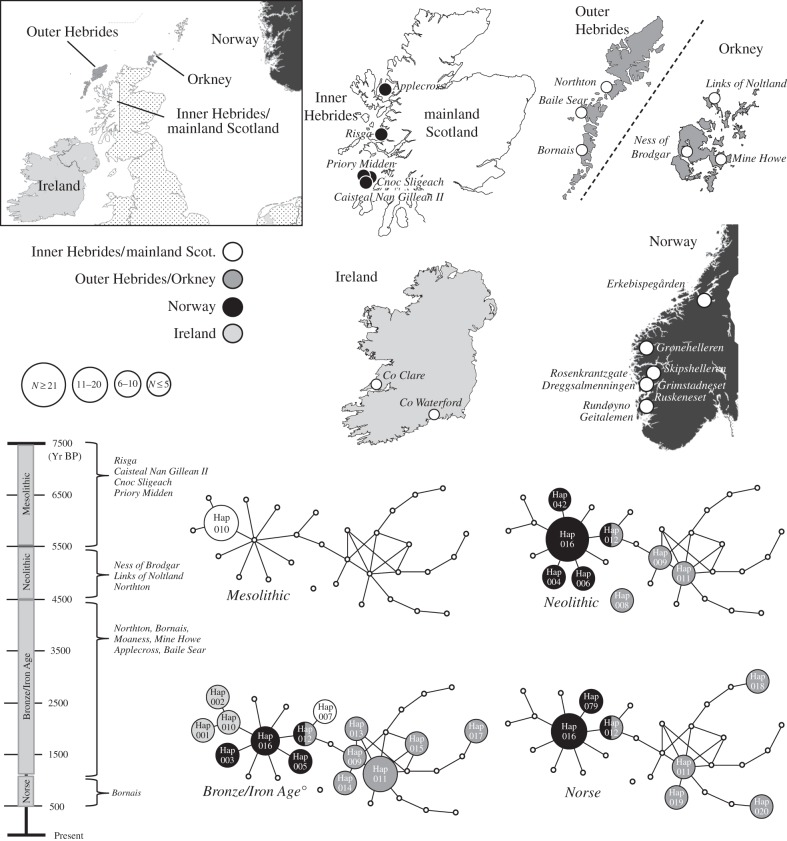
Network constructed using TCS 1.21 of all comparable ancient red deer haplotypes, generated in this study (labelled ‘Outer Hebrides/Orkney’ and ‘Inner Hebrides’) and in previous studies. Haplotype circle size is approximately proportional to sample size, and small hollow circles represent unsampled haplotypes in any given time period. Haplotype Hap008 could not be connected to any other haplotype with greater than 95% confidence by TCS. Details of each haplotype ID is given in electronic supplementary material, table S2. Sampling location is given by colour, which corresponds to the map inserts, on which archaeological sampling sites of the new samples sequenced in this study and of previous studies are shown by circles. A timeline of (our definitions of) the archaeological periods is shown on the left, which also describes the sampling sites (of our new samples) that each of these time periods are represented in.

**Figure 2.**
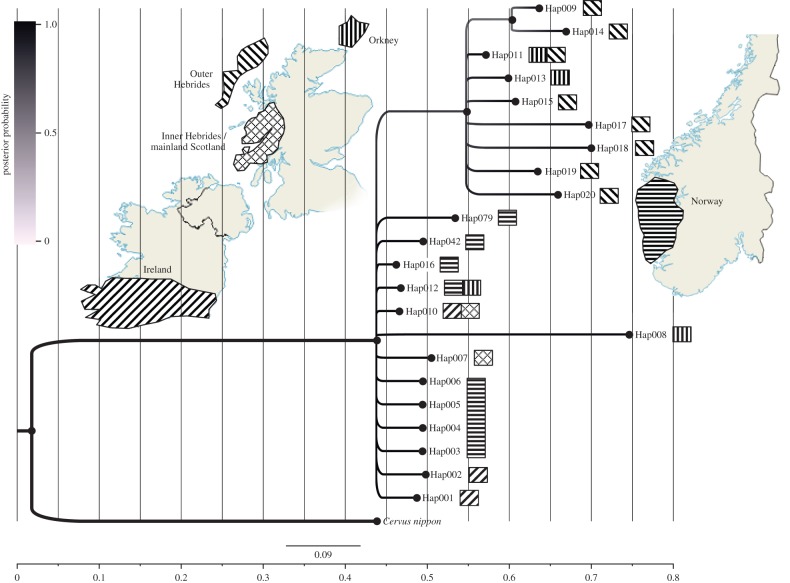
Bayesian phylogeny constructed using MrBayes v. 3.2.2 of all comparable ancient red deer samples, generated in this study and in previous studies, from Ireland, the Outer Hebrides, Orkney, the Inner Hebrides/mainland Scotland and Norway. The tree was rooted with *Cervus nippon* as an outgroup. (Online version in colour.)

Figure 1 features a network of ancient red deer sequences from northern Europe, including available and comparable sequences from previous studies [27,32], as well as the Tyrolean ice-man’ deer sequence [33]. This network demonstrates that the two Inner Hebridean haplotypes cluster with the Irish and Norwegian haplotypes. The most common Inner Hebridean haplotype (Hap010, *n* = 11) was previously found in 2722–3340 cal. yr BP samples from Ireland [8]. All Outer island samples grouped together, except for one haplotype (Hap008, *n* = 2) found in Orkney that was highly distinct and did not cluster with any of the other haplotypes used in this study, based on the 95% confidence cut-off value of TCS. The corresponding Bayesian phylogeny is shown in figure 2 (and corresponding neighbour-joining phylogeny in electronic supplementary material, figure S1), which grouped samples from the Outer Hebrides and Orkney on an independent branch (posterior probability 0.76), separate from the remaining samples (Norway, Ireland, Inner Hebrides and mainland Scotland). A table of haplotype frequencies and locations is given in electronic supplementary material, table S3.

### (c) Ancient deer in the Scottish Isles

When considering only the ancient Scottish samples sequenced in this study, mtDNA genetic diversity was found to be partitioned by both time period and geographical origin. For example, the Mesolithic (*n* = 12, no. haps = 2), Neolithic (*n* = 8, no. haps = 4), Bronze/Iron age (*n* = 22, no. haps = 8) and Norse (*n* = 4, no. haps = 4) time periods had 100%, 50%, 75% and 75%, respectively, of their haplotypes novel to that time period. We also found that genetic diversity of time periods increased towards the present day. Haplotype diversity was low in Mesolithic samples (0.167), and monotonically increased throughout the time periods investigated (Neolithic, 0.750; Bronze/Iron Age, 0.944; Norse, 1.000). The low haplotype diversity in the Mesolithic samples corresponds to two haplotypes, with one of these being found in only one sample. Our data also showed high and significant *F*_ST_ values between the Mesolithic versus all other time periods (*F*_ST_ = 0.525–0.780, *p* < 0.001). The only other significant pairwise *F*_ST_ value was between the Neolithic and Iron/Bronze age samples (*F*_ST_ = 0.230, *p* < 0.001; table 2). The most common haplotype in our dataset (H011, *n* = 20) was found throughout all time periods, except the Mesolithic. The second-most common haplotype (H010, *n* = 11) was found exclusively in the Mesolithic and none of the haplotypes found in the Mesolithic were found in any other time period. Our dataset contained 14 haplotypes, from the Inner Hebrides (*n* = 3), Outer Hebrides (*n* = 8), Orkney (*n* = 2), and both Orkney and the Outer Hebrides (*n* = 1). Haplotype diversity was highest in the Outer Hebrides (0.957), followed by Orkney (0.600) and then the Inner Hebrides (0.423). There was significant genetic differentiation among all regional comparisons. This differentiation was particularly high between the Outer and Inner Hebrides, and between Orkney and the Inner Hebrides, demonstrated by very high (*F*_ST_ = 0.667 for Outer versus Inner Hebrides and 0.491 for Orkney versus Inner Hebrides), and significant (*p* < 0.001) pairwise *F*_ST_ values, between samples from the Inner Hebrides and the other two regions (table 3). Within sampling period *and* location, haplotype diversity was 0.167 for the Inner Hebrides Mesolithic, 0.400, 0.540 and 1.000 for Outer Hebrides Neolithic, Iron/Bronze Age and Norse, respectively, and 0.667 and 0.000 for Orkney Neolithic and Iron/Bronze Age, respectively.

**Table 2.** Pairwise *F*_ST_ values for time period.

	Mesolithic	Neolithic	Iron/Bronze Age
Neolithic	0.525***	/	/
Iron/Bronze Age	0.754***	0.230***	/
Norse	0.780***	0.111^n.s.^	0.098^n.s.^

**Table 3.** Pairwise *F*_ST_ values for geographical region.

	Outer Hebrides	Orkney
Orkney	0.095*	/
Inner Hebrides/mainland	0.667***	0.491***

### (d) Modern and ancient genetic diversity

**Figure 3.**
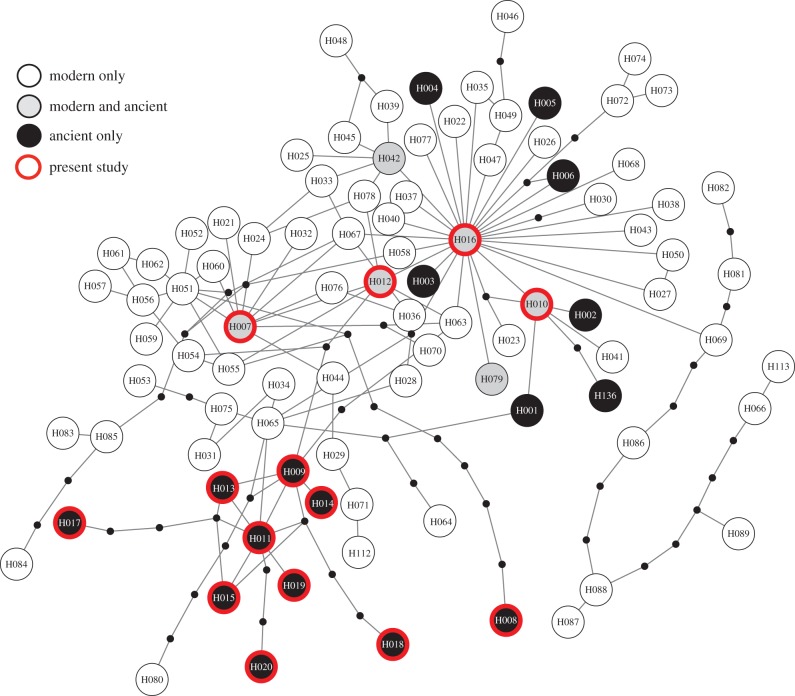
Network constructed using TCS 1.21 of all comparable modern and ancient red deer haplotypes, generated in this study (with thick outline) and in previous studies. Haplotype circle size is not proportional to sample size, as frequencies were not available for all haplotypes and/or locations. Small, black circles correspond to unsampled haplotypes. Details of each haplotype ID is given in electronic supplementary material, table S2. Geographic location of each haplotype is shown in electronic supplementary material, figure S2. (Online version in colour.)

Both outer island and Inner Hebridean ancient deer samples clustered with the Western lineage of European deer, but 10 of our 14 haplotypes were novel to this study. These 10 novel haplotypes were all found in the outer islands, and constituted all but one of the outer island haplotypes detected. As is the case for the ancient samples in figure 1, the network of all mtDNA sequences (figure 3) groups Inner Hebridean and mainland Scotland deer with modern and ancient samples from Scotland, Ireland and Norway. All outer island deer sequences generated here clustered close to, but separate from the other northern European samples, except for one haplotype (H012) that matched sequences previously found in ancient Norwegian [20] and modern mainland Scottish [34] deer. This sequence also matched that of the deer hair in the clothing of the Copper Age human found in the Italian Alps [21]. The Bayesian phylogeny of all modern and ancient samples (electronic supplementary material, figure S2) was less clear due to poor resolution of some branches. However, as with the network, all ancient samples that formed clades independent from the modern samples were from the outer isles (Hap009, 014, 017 and 020).

## 4. Discussion

Unexpectedly, our data showed that outer island ancient Scottish red deer were unlikely to have originated from mainland Scotland. We found no shared haplotypes between the ancient outer isle and Inner Hebridean/mainland deer, and higher genetic diversity in the former, relative to the latter, with Mesolithic Inner Hebridean/mainland Scotland deer having the second-lowest genetic diversity of any sample grouping. In addition, very strong genetic differentiation between both the Outer Hebrides versus Inner Hebrides and mainland, and Orkney versus the Inner Hebrides and mainland, was found, implying a lack of gene flow between these regions. It therefore seems likely that the Outer Hebrides and Orkney deer that appeared in the Neolithic were not colonized by Scottish Mesolithic mainland or Inner Hebridean deer, but instead from a location that has not previously been considered.

It has previously been suggested that the outer Scottish Isles are located too far from mainland Scotland for them to have been colonized naturally by red deer [16]. Our results support this hypothesis, but also indicate that Neolithic humans introduced red deer into the outer Scottish Isles from an unknown origin of a greater distance than previously suggested. It is possible that (because we did not have Neolithic samples from mainland Scotland) the outer isle deer originated from a Neolithic mainland Scotland population that we did not sample. However, our Mesolithic samples originated from four separate sites across the Inner Hebrides and mainland Scotland (suggesting that they are representative of the region), and the haplotypes detected have all been found in modern Scottish deer (implying that these haplotypes persisted through time until the present), whereas nine out of the ten outer isle haplotypes were unique to this study. Our results therefore imply long-distance maritime travel, and associated transport of livestock, by humans during the Neolithic. In Europe, short-distance maritime transport of deer (Megaloceros) has been identified from as early as approximately 24 000–20 500 yr BP in the Mediterranean [6]. Further north, it is thought that humans introduced red deer into Ireland during the Neolithic Bronze Age [8], and transported the Orkney vole from mainland Europe to Orkney (at least 5100 yr BP [4]). Interestingly, Martínková *et al.* [4] posit that the large number of voles that must have arrived on Orkney imply the transport of livestock from mainland Europe (Belgium) with the vole ‘stowaways’ in the grass, bedding or fodder. That study found high genetic diversity of voles, indicating that a relatively large number of individuals must have been moved. This study finds relatively high genetic diversity in Neolithic deer from Orkney and the Outer Hebrides, also suggesting that a large number of individuals may have been moved to establish those populations. Our results do not identify any obvious source population, although the outer island deer cluster with the Western lineage of European deer, so the source is likely to be northern Europe. An alternative explanation to long-distance maritime travel is that our genetically distinct Neolithic red deer represent the descendants of a population of deer that inhabited a northern refugium during the LGM that were replaced by southern immigrants during the Holocene. Meiri *et al.* [10] identified a highly distinct mitochondrial haplotype, which they hypothesized could have arisen in a Northern refugium. However, there is currently no evidence that red deer persisted in Britain during the LGM [35].

While deer form a major component of Mesolithic assemblages across Britain, post-Mesolithic they comprise an insignificant proportion (around 1%). From the Neolithic onward they only continued to be exploited in significant quantities on the islands, and the proportion of samples that are deer varies across time, space and type of site. For example, they comprise two-thirds of the material within Mesolithic Oronsay and up to one-quarter of later prehistoric tomb assemblages on Orkney, with similar proportions in Norse sites on Lewis/Harris. On other sites they are only present in small, but persistent proportions (e.g. 3–6% on South Uist Norse sites [16]). As noted above, red deer are not reported from the Outer Hebrides and Orkney until the Neolithic period and are associated with tombs and settlements on Orkney [16]. The early populations were notably smaller than contemporary mainland deer and this could be a product of the introduction of already size diminished species (due to insular dwarfism [36]). Interestingly, the majority of Mesolithic deer from the tiny Inner Hebridean island of Oronsay are of a similar size to mainland Scottish and English deer [16], but a minority are approximately 30% smaller. As Oronsay is considered too small to host an endemic population, these smaller deer may originate from a population living on another, larger island, with the larger animals representing imported mainland individuals [37].

The Inner Hebridean and mainland samples shared common haplotypes with modern samples from mainland Scotland and the Inner Hebrides, and ancient Irish and Norwegian red deer. In the case of ancient samples, this result suggests gene flow between and/or common colonization history between these regions (Inner Hebrides, mainland Scotland, Ireland and Norway). This could be explained by the colonization of these regions via a single rapid expansion from a glacial refugium, as previously suggested [10]. There is a strong concordance between haplotypes from mainland Scotland/Inner Hebrides and Ireland, reinforcing previous conclusions that Ireland was first colonized from Britain following the LGM [8], and indicating that the colonization route may have been via the Inner Hebrides. In the case of the modern samples, our results suggest that ancient maternal lineages of red deer have, to some extent, persisted in mainland Scotland and the Inner Hebrides to the modern day, although there has undoubtedly been a contribution of foreign deer into the gene pool [18,33,38]. We found a common haplotype across an Orcadian and two Outer Hebridean sites, and relatively low genetic differentiation, demonstrating either common ancestry between these islands, or gene flow across the outer Scottish islands from the Neolithic onwards (figure 1). This association between the outer Scottish Isles implies that their colonization by red deer was due to a common biogeographic or anthropogenic process.

It is known that there were Scandinavian settlers and visitors across the Scottish Isles from approximately the ninth century onwards [16]. This makes it possible that deer from Scandinavia or other regions supplemented insular Scottish deer populations during the Norse period. Assemblages from mainland Scotland and the Inner Hebrides containing red deer are scarce during this time, and there is little evidence for any endemic deer population in Orkney from the Norse period onwards. As a result, our Norse samples were restricted to the Outer Hebrides. The majority of the haplotypes found in the Norse samples were novel to that particular time period (75%), and this was also the time period with the highest genetic diversity. There was a monotonic increase in haplotype diversity through the time periods investigated. This finding, in combination with the high genetic differentiation when Mesolithic sites are included in pairwise comparisons, compared with comparisons that do not include the Mesolithic sites, suggests an increase in gene flow since the Mesolithic. An increase in gene flow and genetic diversity from the Neolithic onwards is consistent with human-mediated translocations of red deer, introducing a large number of red deer with novel haplotypes into the Scottish islands. However, none of the novel haplotypes that we found during the Norse period matched any haplotypes detected in any other regions by previous studies, including in Scandinavia. Also, the increase in diversity over time did not hold true for all sampling regions when they were assessed independently. In addition, all time periods contained novel haplotypes, and our sample size from the Norse period was very low (*n* = 4), which makes it very difficult to make conclusions about demographic processes such as immigration or population expansions.

There is an on-going debate on the movement of human populations during the Mesolithic and Neolithic, with both indigenous adoption and colonization suggested for the introduction of farming [39]. Archaeological lithic, ceramic and monumental evidence points towards contact with France and the Low Countries, indicating movement both across the channel and up the west coast [40,41]. At present, there is limited faunal ‘sourcing’ information available for the movement of either domesticated or wild species, apart from the putative low country source for the introduced Orcadian vole [4]. This research presents the first attempts to understand the deliberate translocation of faunal species into insular Britain and track the source for these introductions.

One haplotype found in our outer island samples has previously been detected in ancient Norwegian deer [20], modern Scottish deer [34] and from a deer hair found on the clothing of a Copper Age human, known as the Tyrolean Iceman, or Ötzi (dated at 5350–5100 yr BP), from the Italian Alps [21]. This haplotype therefore appears to be ancestrally widespread; however, the haplotype is also found in locations known to have been founded via human introductions (the outer Scottish Isles; this study), and has previously been associated with ancient human remains [21]. The distribution of this haplotype is therefore likely to be a result of both natural expansion as well as ancient human translocations of red deer. Our results show that modern red deer translocations can confound the genetic signatures of original and ancient colonization events, validating the use of aDNA to investigate this question. Future work should (i) focus on aDNA from Mesolithic deer sites from mainland England (and Scotland where possible) in order to further contextualize the Inner Hebridean deer, (ii) extend aDNA sampling throughout Europe, and (iii) increase the sampling effort (both number of samples and amount of sequence information) from the Neolithic Outer Hebrides and Orkney deer. Modern DNA of Western Isles (Inner and Outer Hebrides) deer would also be useful for reconstructing historical demography.

Our results suggest human-mediated introduction of red deer to the outer Scottish Isles from a currently unidentified source population. Along with Carpathian deer [42], Outer Hebridean deer are one of the least managed populations remaining, although their initial introduction to these islands was clearly originally facilitated by humans. This has important conservation implications, reinforcing previous recommendations that the value of Britain's remaining insular deer should not be underestimated [16]. Red deer are no longer present on the Northern Isles (Orkney and Shetlands), but this study shows that ancient populations of Western and Northern Isles deer may have been closely related in the past. This suggests that if reintroductions of deer onto the Northern Isles became a possibility, the Outer Hebrides would be a strong candidate for the source population.

## Supplementary Material

Figure S1

## Supplementary Material

Figure S2

## Supplementary Material

Supplementary methods S1

## Supplementary Material

Table S1

## Supplementary Material

Table S2

## Supplementary Material

Table S3

## Data accessibility

All new sequences generated in this study have been deposited in GenBank, accession numbers: KU877699–KU877712. The datasets supporting this article have also been uploaded as the electronic supplementary material.
