# Supplementary figures and images for: Colonization of the Scottish islands via long-distance Neolithic transport of red deer (Cervus elaphus)
Source: Proc Biol Sci. 2016 Apr 13;283(1828):20160095. doi: 10.1098/rspb.2016.0095 (PMC4843653; doi:10.1098/rspb.2016.0095)

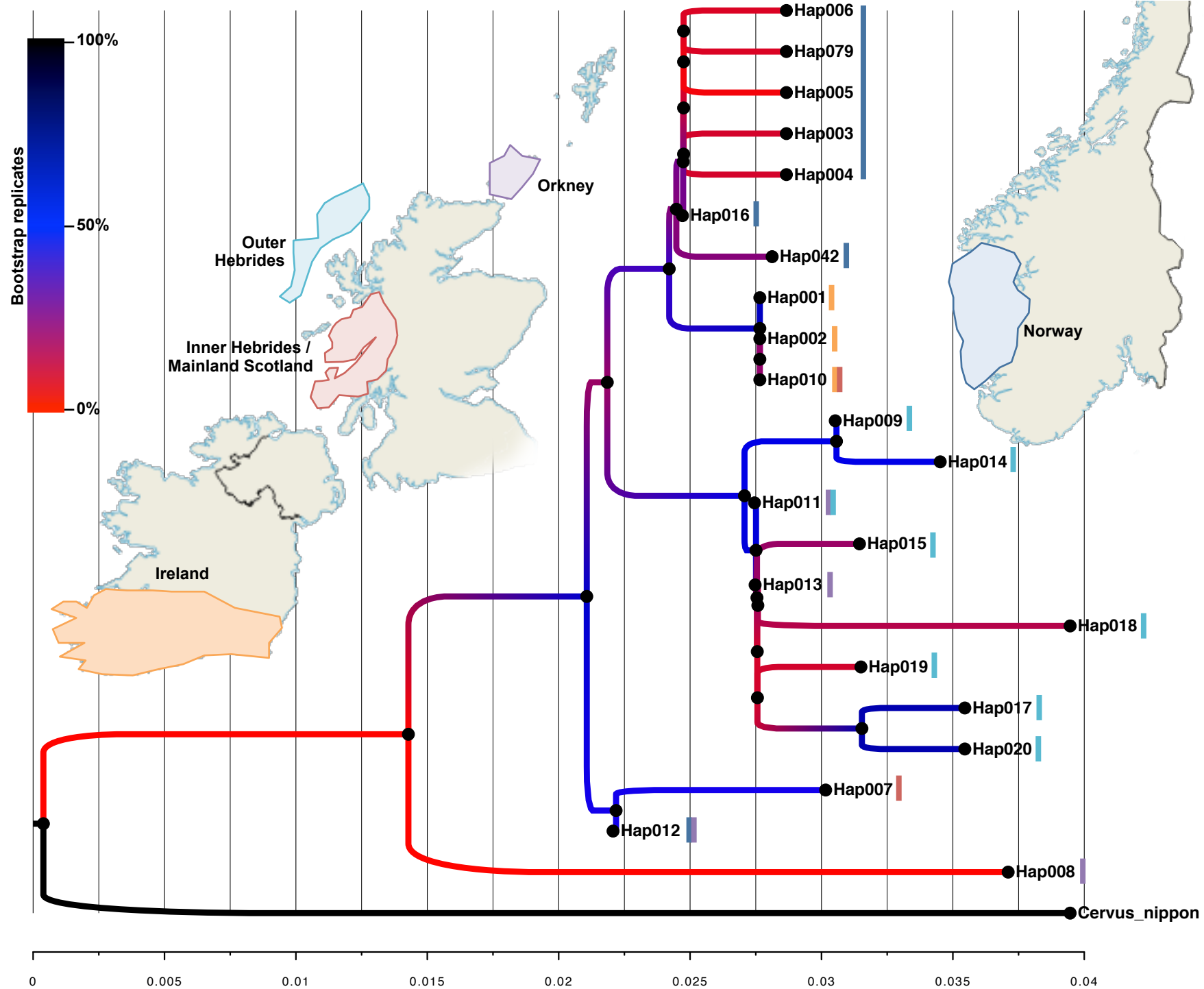

Supplement: Figure S1 [file rspb20160095supp1.pdf]

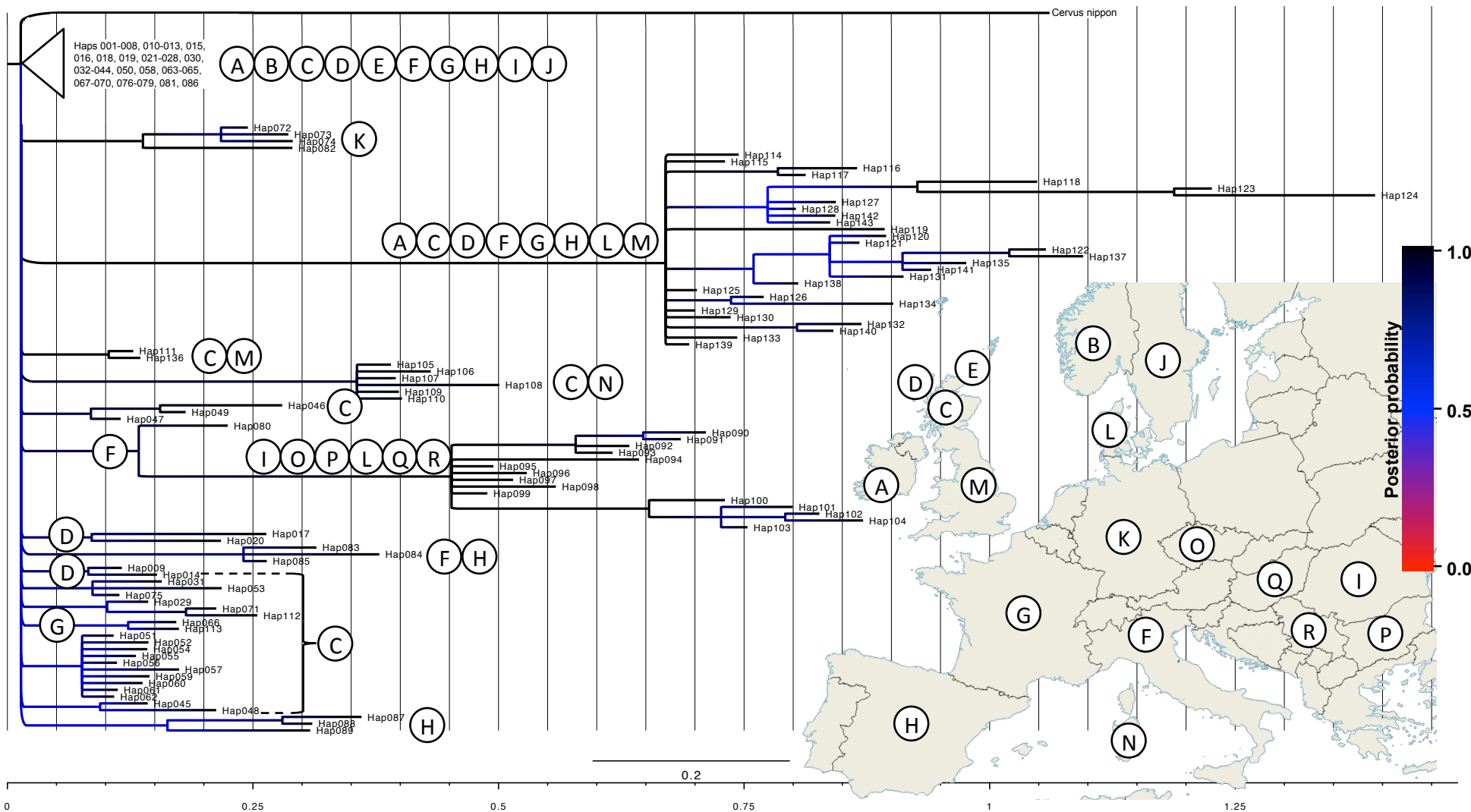

Supplement: Figure S2 [file rspb20160095supp2.pdf]
