# Supplementary material for: Colonization of the Scottish islands via long-distance Neolithic transport of red deer (Cervus elaphus)
Source: Proc Biol Sci. 2016 Apr 13;283(1828):20160095. doi: 10.1098/rspb.2016.0095 (PMC4843653; doi:10.1098/rspb.2016.0095)
Supplement: Supplementary methods S1 [file rspb20160095supp3.docx]

**Supplementary methods S1**

**Definition of time periods**

Archaeological time periods were classified as follows:

- Mesolithic, 7,500 – 5,500 cal. Yr BP (1)
- Neolithic, 5,500 – 4,500 cal. Yr BP (2)
- Bronze/Iron Age 4,500 – 1,100 cal. Yr BP (3, 4)
- Norse, 1,100 – 500 cal. Yr BP (4, 5)

**Additional sequences**

Our samples were compared to all available sequences from the literature. The samples that these references correspond to is given in table S2:

- Skog *et al.* (2009); (6)
- McDevitt *et al.* (2009); (7)
- Nussey *et al.* (2006); (8)
- Carden *et al.* (2012); (9)
- Fickel *et al.* (2012); (10)
- Hmwe *et al.* (2006); (11)
- Nielsen *et al.* (2008); (12)
- Pérez-Espona *et al.* (2009); (13)
- Randi *et al.* (2001); (14)

**Ancient DNA protocol**

For each set of DNA extractions (maximum of 10 samples) we carried out two extraction negatives. For the first extraction negative we vortexed 15 ml of the extraction buffer and Proteinase K solution in the freezer mill tubes (including the metal hammer and plugs) and then proceeded with the extraction as if this extraction buffer was a dissolved bone sample. For the second extraction negative, we did the same, excluding the stage where the buffer was vortexed in the extraction tube. These two extraction negatives were designed to (in the case of detecting contamination) be able to differentiate between contamination originating during or after the physical break-up of the samples. We also carried out at least two PCR negatives for each extraction set.

**References**

1. Saville, A and Wickham-Jones, C (2012) Palaeolithic and Mesolithic Scotland: ScARF Panel Report Panel Report. June. Available from: http://tinyurl.com/d86dgfq

2. Sheridan A, Brophy K. (2012) Neolithic Scotland: ScARF Panel Report. June. Available from: http://bit.ly/1Nuz3PY

3. Downes J. (2012) Chalcolithic and Bronze Age Scotland: ScARF Panel Report. June. Available from: http://tinyurl.com/clxgf5s.

4. Hall, M and Price, N. (2012) Medieval Scotland: A future for its Past. June. Available from: http://tinyurl.com/bogzn5w

5. Hunter F, Carruthers M. (2012) Iron Age Scotland: ScARF Panel Report. June. Available from: http://tinyurl.com/cx4nlt8

6. Skog A., Zachos FE, Rueness EK, Feulner PGD, Mysterud a., Langvatn R, et al. Phylogeography of red deer (*Cervus elaphus*) in Europe. J Biogeogr. 2009 Jan; 36 (1): 66–77.

7. McDevitt AD, Edwards CJ, O’Toole P, O’Sullivan P, O’Reilly C, Carden RF. Genetic structure of, and hybridisation between, red (*Cervus elaphus*) and sika (*Cervus nippon*) deer in Ireland. Mamm Biol. 2009; 74 (4): 263–73.

8. Nussey DH, Pemberton J, Donald a, Kruuk LEB. Genetic consequences of human management in an introduced island population of red deer (*Cervus elaphus*). Heredity (Edinb). 2006; 97 (1): 56–65.

9. Carden RF, McDevitt AD, Zachos FE, Woodman PC, O’Toole P, Rose H, et al. Phylogeographic, ancient DNA, fossil and morphometric analyses reveal ancient and modern introductions of a large mammal: The complex case of red deer (*Cervus elaphus*) in Ireland. Quat Sci Rev [Internet]. Elsevier Ltd; 2012; 42: 74–84. Available from: http://dx.doi.org/10.1016/j.quascirev.2012.02.012

10. Fickel J, Bubliy OA, Stache A, Noventa T, Jirsa A, Heurich M. Crossing the border? Structure of the red deer (*Cervus elaphus*) population from the Bavarian-Bohemian forest ecosystem. Mamm Biol [Internet]. Elsevier GmbH; 2012; 77 (3): 211–20. http://dx.doi.org/10.1016/j.mambio.2011.11.005

11. Hmwe SS, Zachos FE, Sale JB, Rose HR, Hartl GB. Genetic variability and differentiation in red deer (*Cervus elaphus*) from Scotland and England. J Zool. 2006; 270 (3): 479–87.

12. Nielsen EK, Olesen CR, Pertoldi C, Gravlund P, Barker JSF, Mucci N, et al. Genetic structure of the Danish red deer (*Cervus elaphus*). Biol J Linn Soc. 2008; 95 (4): 688–701.

13. Pérez-Espona S, Pérez-Barbería FJ, Goodall-Copestake WP, Jiggins CD, Gordon IJ, Pemberton JM. Genetic diversity and population structure of Scottish Highland red deer (*Cervus elaphus*) populations: a mitochondrial survey. Heredity (Edinb). 2009; 102 (2): 199–210.

14. Randi E, Mucci N, Claro-Hergueta F, Bonnet A, Douzery EJP. A mitochondrial DNA control region phylogeny of the Cervinae: speciation in *Cervus* and implications for conservation. Anim Conserv. 2001; 4(1): 1–11.
